# Empiricism

**Published:** 1883-07

**Authors:** J. Taft


### COMMUNICATIONS.

### Empiricism.

BY J. TAFT.

[Read before the Mad River Dental Society, May 22, 1883.]

"What should be done further to protect the public from the increasing empiricism practiced in our large cities?"

The question assumes that empiricism is on the increase, especially in the large cities, and it would seem to imply that it exists to a greater extent, and is increasing more rapidly, in the large cities than in the smaller cities, towns, and country. This is certainly not self-evident, and upon a careful survey, it fails to be established. The fact is, empiricism abounds everywhere, to a greater extent in some localities than others, depending quite as much upon the character of the population, for its growth, as anything else. In communities that are pecuniarily prosperous, ignorant, penurious, and proud, a fertile field exists for the display and growth of this evil.

This is true, whether of cities, towns, or country—wherever the conditions exist for the propagation and growth of quackery, it will certainly be found. Let it be borne in mind however, that the people of a community may be intelligent upon many subjects, yes, upon all the general topics of the day and times, and yet exceedingly ignorant upon that which pertains to the laws of life, health, and physical well-being. Such persons are often quite as easily imposed upon as the most ignorant, and manifest a stupidity in reference to the teeth, and the efficiency and skill of the dentist, that is surprising, and altogether inconsistent with their general intelligence.

How often is it that men, and women too, of education and culture, ministers, judges, esquires, colonels, majors, senators, occasionally lawyers, and too often physicians will not only patronize, but endorse and recommend the most arrant quacks and quackery. To be fully assured of this, it is only necessary to consult the advertisements of quacks and nostrums usually endorsed by the classes mentioned above in the newspaper-press of the country--daily, weekly, monthly, religious, secular, professional, scientific, etc. There are a few papers, half a dozen, more or less perhaps of the thousands of papers in the country who claim to have freed their pages of this iniquity, but they are exceedingly rare.

There's millions in it.

The word "further," in the question conveys the idea that something has been done to counteract empiricism in the past. That the relative empiricism of the present is less than that of the past is a question concerning which there may be differences of opinion, but that the amount of quackery, both absolute and relative, is far less than it would have been in the absence of certain instrumentalities that have been in operation, no one can deny.

The following four agencies have been the chief factors in the development, growth and establishment in its present position, at least, of the art, science and profession of dentistry, viz., *Associative effort*; 2. *A literature, both standard and journalistic*; 3. *Collegiate education*; 4. *Legal enactments.*

It would seem that the bare mention of these instrumentalities would be sufficient to bring clearly to the minds of all present the influence that each has had in the upbuilding of our profession. When the combined influences of these agencies exist, it is impossible that empiricism can flourish. As men become educated, they become less inclined towards engaging in the doubtful practices of the quack. Our literature is a potent agency in education, not only for the student, but for the practitioner as well, and how important is it, that the text books put into the hands of students be such as will give them correct ideas and a just apprehension of that which they are to learn. Very rarely, if at all, will he who has attained a thorough knowledge of Anatomy, Physiology and Chemistry, become an empiric in any department of medical practice. How all important then that the foundation of the professional attainments of the dentist be laid deep and broad.

That there should be better arranged text books for the preparatory work of the student, I think no one will deny.

Our periodical literature, which is designed more especially for the practitioner, should have for its aim, not only the communication of facts, modes, operations and news, but it should also in a large measure present and discuss the principles upon which the practice of the profession is based, and this should be done in such a manner as to elicit attention and secure the earnest interest of those who read. The persistent student never becomes a quack.

In view of the capabilities of our periodical literature, and the magnitude and importance of the work it may accomplish, how very desirable it is that those engaged in this department should work harmoniously—co-operate in all ways possible for the accomplishment of the greater good; engagement in a common cause should induce a common sympathy, and a co-operation of effort. Every journal should be a light-bearer, a disseminator of knowledge.

Self conceit or vaunted superiority is certainly not an essential part of true journalism.

How to increase the number of readers of our journals is a problem worthy of especial consideration. A very small proportion of the profession are careful readers of our dental periodicals—probably not more than one in ten. There is, without doubt, a much larger number who scan the journals for items of a practical sort, but who never think of studying the matters of deeper import, which require thought and patience for their comprehension and appropriation. Another class of persons and probably comprising a greater number than either of those referred to, are those who usually confine their reading to the advertising pages. The latter two classes, and especially the last one, usually receive their journals as a gratuity. No dentist, who has subscribed and paid for from two to four good dental journals, and carefully reads them, can be a quack.

The education of those, who propose to enter the ranks of the profession, is fraught with more importance than is usually attached to it. There are two ways in which this is accomplished, viz., in the office and in the college. It is ordinarily accounted a light matter to have in charge a dental student. Many young men make it a point, soon, sometimes immediately, after opening an office to take a student, imagining that thereby their own importance and prestige will be promoted; while the truth is that the ability to teach another is usually the least of his attainments. Many students are injured, if not permanently spoiled, by such puerile instruction. But the young practitioners by no means do all the mischief in this direction. Men who have been many years in practice, in many instances, are totally unfit to train students; they either have not the natural endowments, or the acquired ability, or the industry, and perhaps not the time or strength. Now all this may exist, and only result in a negative injury. But in too many instances a positive mischief is done, either by the impartation of false notions as to what constitutes true professional character, or by erroneous teaching of principles and practices.

There will not be found more than one in an hundred who has been in practice more than ten years, who can or will properly instruct a dental student.

It is often found that those who enter a dental college, after receiving office pupilage, are the most difficult to set right, and the most unpromising throughout the course of instruction. Persons possessing no natural aptitude for dental practice are too often encouraged to enter upon a course of preparation for it, and the outcome in such cases generally is, either a total failure, or an awkward bungling mode of practice, or down-right empiricism; the latter more often than otherwise.

The regular well-ordered and systematic course of instruction in a dental college renders it almost impossible that the recipient should be a quack. It is the province of the college to impart to those seeking its advantages, a thorough knowledge of its principles, and the practice of its science and art, and he who is well grounded in these will not descend to the ways of the charlatan; the half or poorly educated are the more likely to enter upon "ways that are dark and tricks that are vain."

It must be borne in mind, however, that it is not always in the power of any college faculty to convert a rogue into an honest man.

But it is, nevertheless, the duty of those who are preparing actors for important work and great responsibilities, to look to all the existing qualities, moral as well as intellectual, that may in any wise impair efficiency and success, and correct defects and perversities as far as possible. Good example will do much in this respect, but precept upon precept and line upon line should not be omitted when needed.

In the reception of students by our colleges, it would be well if a proper system of discrimination could be adopted by which the manifestly unfit and incapable would be prevented from entering; the evils resulting from want of proper discrimination in this respect have been too often manifest.

Notwithstanding the disabilities under which our colleges have labored, they have stood as a great barrier to, and a prevention of empiricism.

It is doubtful if any other single agency has done more, certainly none has laid deeper or broader foundations. But the suggestion is here ventured that by the exercise of proper and judicious co-operation, far greater and better results could be attained than hitherto. Were our colleges to present a united and unyielding front, quackery would flee away and hide, if it existed at all, in the dark places of the earth.

The unjustifiable and pernicious emulation and even strife, that has so often been employed to secure students, should be abandoned and an emulation as to who should accomplish the most thorough work in the education of students be substituted. Such a course would at once elevate the dignity and efficiency of our educational system; it would give a greater degree of strength, and command more profound respect; then, instead of being in a begging attitude to the rank and file of the average students, many of whom are mercenary and unprofessional in their aspirations and intuitions; the colleges could establish rules and regulations to serve the best interests of all concerned, and carry out their work accordingly. Such a course would place a broad and heavy heel upon quackery in the city, in the town, and in the country.

Within the past few years, legal enactments have been brought into existence and put into operation for the suppression and prevention of empiricism, and for stimulating to a higher degree of professional attainments. This is the most recent of the four agencies mentioned in the former part of this paper, and so has not the advantage of so long a test of its efficiency as the others, still it has so well proven its capability that a large number of the States of the Union have enacted laws "regulating the practice of dentistry."

They have for the most part proved eminently satisfactory, especially those of more recent enactment, the defects of those first adopted having been corrected in those of recent introduction.

In most States the promulgation of these laws has been the occasion of an exodus, more or less marked, of those whom the law pronounced incompetent.

Where such laws exist, the profession should see that they are enforced so far as possible, and when they are found to be defective or inefficient, seek to have them amended. The laws that have been enacted to regulate the practice of dentistry have been as well executed, if not better, than many others that have been of much longer standing, and in which a far greater number of persons are directly interested.

